# Anomalous sub-diffusion of water in biosystems: From hydrated protein powders to concentrated protein solution to living cells

**DOI:** 10.1063/4.0000036

**Published:** 2020-10-12

**Authors:** Rui Li, Zhuo Liu, Like Li, Juan Huang, Takeshi Yamada, Victoria García Sakai, Pan Tan, Liang Hong

**Affiliations:** 1 School of Physics and Astronomy, Shanghai Jiao Tong University, Shanghai 200240, China; 2 Institute of Natural Sciences, Shanghai Jiao Tong University, Shanghai 200240, China; 3 Institute for Advanced Study, The Hong Kong University of Science and Technology, Hong Kong, China; 4 Zhiyuan College, Shanghai Jiao Tong University, Shanghai 200240, China; 5 School of Life Sciences and Biotechnology, Shanghai Jiao Tong University, Shanghai 200240, China; 6 Neutron Science and Technology Center, Comprehensive Research Organization for Science and Society, Ibaraki 319-1106, Japan; 7 ISIS Facility, Rutherford Appleton Laboratory, Chilton, Didcot OX11 0QX, United Kingdom

## Abstract

Water is essential to life and its translational motion in living systems mediates various biological processes, including transportation of function-required ingredients and facilitating the interaction between biomacromolecules. By combining neutron scattering and isotopic labeling, the present work characterizes translational motion of water on a biomolecular surface, in a range of systems: a hydrated protein powder, a concentrated protein solution, and in living *Escherichia coli* (*E. coli*) cells. Anomalous sub-diffusion of water is observed in all samples, which is alleviated upon increasing the water content. Complementary molecular dynamics simulations and coarse-grained numerical modeling demonstrated that the sub-diffusive behavior results from the heterogeneous distribution of microscopic translational mobility of interfacial water. Moreover, by comparing the experimental results measured on *E. coli* cells with those from a concentrated protein solution with the same amount of water, we show that water in the two samples has a similar average mobility, however the underlying distribution of motion is more heterogeneous in the living cell.

## I. INTRODUCTION

Water is an active ingredient in cell biology. Biomacromolecules inside the cell are encapsulated in a shell of hydration water, whose structure and dynamics are distinct from pristine bulk water.^1–7^ This hydration shell of water plays a key role in stabilizing the 3-D structure of the biomolecules^2^ and lubricating them to exhibit the required flexibility for function.^8,9^ Particularly, the diffusive motions of water aid ligand and proton transfer, protein–DNA and protein–ligand recognition, protein dynamical transition, and folding of the protein molecule into the correct 3-D structure.^10–19^

Neutron scattering constitutes a valuable experimental approach to explore the diffusive dynamics of water, as it can furnish simultaneously both spatial and temporal information. Moreover, as neutrons are highly sensitive to hydrogen atoms, whose incoherent scattering cross section is an order of magnitude larger than incoherent/coherent scattering cross section of other elements, one can perform neutron scattering on perdeuterated biosystems hydrated in H_2_O to selectively study the motions of water.^20–27^ This method has been applied to explore the dynamics of water in living cells, which was shown to be highly retarded as compared to bulk water.^23–27^ In particular, a retardation factor of ∼250 was found for water in perdeuterated *Haloarcula marismortui* cells, an extremely halophilic organism originally isolated from the Dead sea.^27^ In addition to neutron scattering, many other experimental techniques, including NMR,^28,29^ optical Kerr effect,^30^ THz spectroscopy,^31^ and ultrafast time-resolved Infrared spectroscopy and dielectric-relaxation spectroscopy,^32^ have also been applied to explore the dynamics of water inside various living cells. Most of these experiments reached a general agreement that the mobility of water is slowed down in the living cell, but to what extent the dynamics of the biological water is perturbed as compared to the bulk water is still debated.^28^ Moreover, neutron scattering experiments were also performed on perdeuterated protein powders hydrated by H_2_O, where only a single layer of water molecules is present on the biomolecular surface, and revealed anomalous sub-diffusive behavior of the surface water, where the mean-squared atomic displacement (MSD) exhibits a fractional power law dependence with time, $\langle x^{2}\left(t\right)\rangle ∼t^{\beta }$, *β* < 1 (Refs. 20, 33, and 34) (*β* = 1 corresponds to Brownian normal diffusion, while *β* > 1 denotes superdiffusive motion^35^). A systematic investigation on how this anomalous diffusion of water evolves from a hydrated protein powder, to a protein solution, to a living cell is lacking, and the microscopic mechanism governing the evolution is unknown.

To this end, we present here results from neutron scattering experiments on perdeuterated proteins hydrated by H_2_O at a series of well-controlled hydration levels, to investigate the translational mobility of water molecules involved. The samples include a hydrated protein powder, a concentrated solution, and perdeuterated *E. coli* cell pellets in H_2_O. We found that the sub-diffusive behavior is generally present in all systems studied, but becomes less prominent upon increasing the water content. Complementary molecular dynamics (MD) simulations and coarse-grained numerical modeling demonstrate that this sub-diffusive behavior results from the heterogeneous distribution of the microscopic translational mobility of water molecules, and the reduction of this heterogeneity upon increasing the water content diminishes the effect. Moreover, by comparing the experimental results measured on *E. coli* cells with those from a concentrated protein solution with the same amount of water, we found that the distribution of water mobility in the living cells is much broader.

## II. RESULTS AND DISCUSSION

**FIG. 1.**
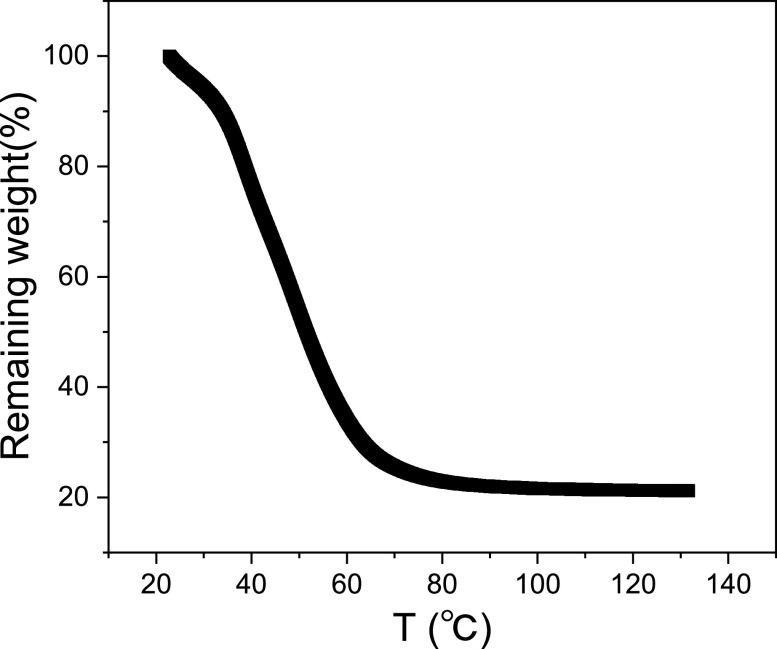
Thermal gravimetric analysis (TGA) result of perdeuterated *E. coli* hydrated in H_2_O.

To examine the water dynamics on a protein surface, we conducted neutron scattering experiments on H_2_O-hydrated perdeuterated Cytochrome P450 protein (CYP) at 280 K at four hydration levels (gram H_2_O/gram protein), i.e., *h* = 0.4, 1.0, 2.0, and 4.0. *h* = 0.4 correspond to a case that the protein surface is covered roughly by a single layer of water molecules,^36^ while *h* = 4.0 denotes multilayers of surface water,^37,38^ corresponding to a concentrated solution. We also measured perdeuterated *E. coli* cell pellets hydrated in H_2_O, where the mass ratio of water in the cells is about 80%, as determined by thermal gravimetric analysis (TGA)^39^ (see Fig. 1), the same as that in the concentrated protein solution at *h* = 4.0. Quasi-elastic neutron scattering data were collected using a range of backscattering spectrometers at different neutron spallation sources around the world, which provide energy resolutions on the order of *μ*eV, probing dynamics in the time window from a few picoseconds to hundreds of picoseconds. Protein samples at *h* = 0.4 and 1.0 were measured on the BASIS instrument at the Oak Ridge National Laboratory in the U.S.,^40^ and those with *h* = 2.0 and 4.0 were measured on the OSIRIS spectrometer at the ISIS Neutron and Muon Source in the UK.^41,42^ Bulk pristine water was examined on the IRIS spectrometer at ISIS and the *E. coli* cells were measured on the DNA spectrometer^43^ at J-PARC in Japan. To complement the experimental data, all-atom molecular dynamics (MD) simulations were performed for the protein samples at the same hydration levels and temperature as the experiments. More detailed information on sample preparation, experimental setup, and MD protocols is provided in the supplementary material.

## III. CHARACTERIZATION OF WATER DYNAMICS BY NEUTRON SCATTERING EXPERIMENTS

**FIG. 2.**
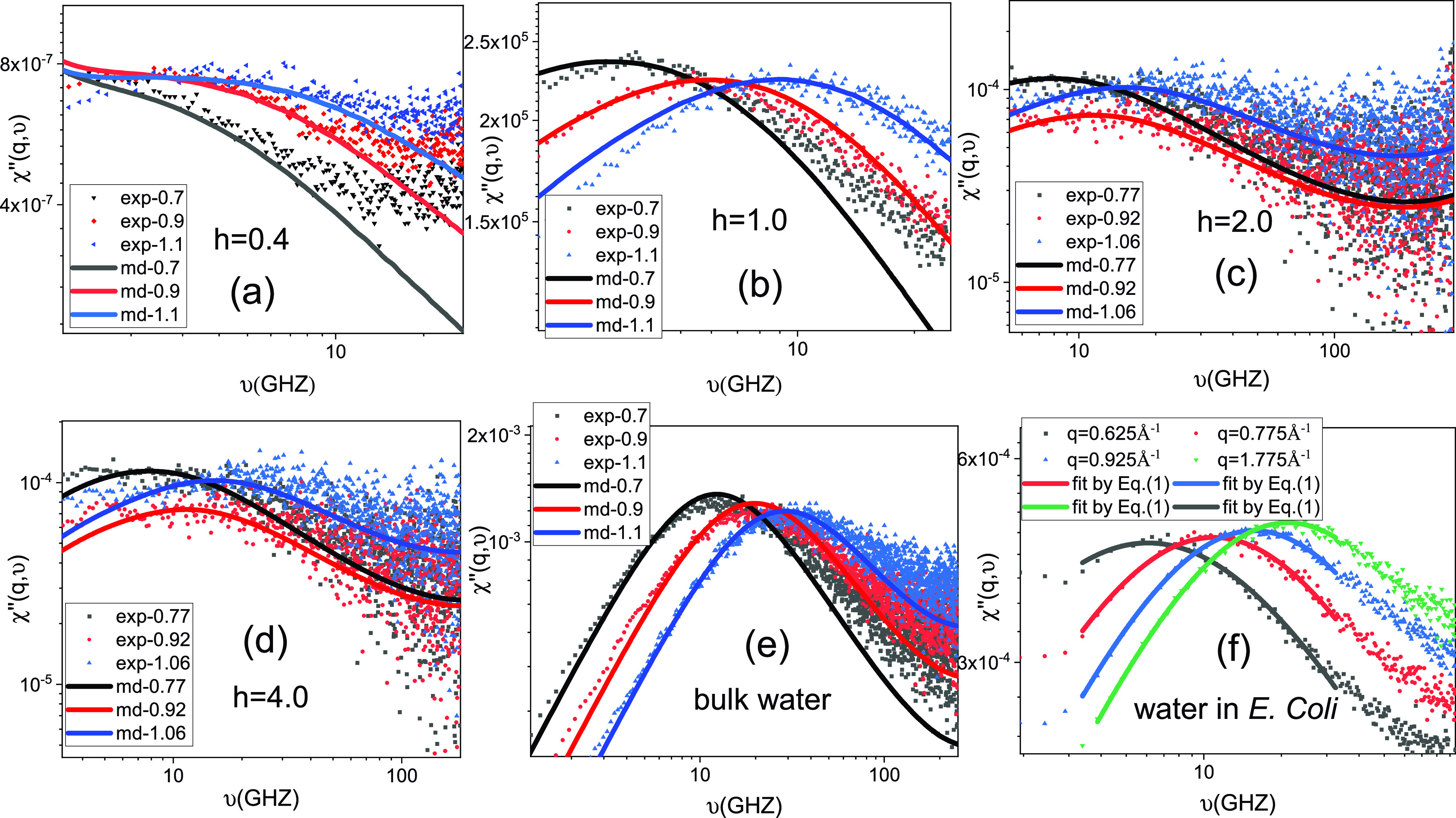
(a)–(e) Neutron susceptibility spectra χ″(q,υ) derived from the experiment and MD simulation on perdeuterated CYP for *h* = 0.4, 1.0, 2.0, and 4.0 and bulk water at different *q*. (f) χ″(q,υ) of water molecules in *E. coli* cells and the corresponding fitting curves using the Cole–Cole distribution function [Eq. (1)] at different *q*-values.

We present the neutron spectra in the form of susceptibility, $\chi^{\prime \prime }\left(q,\;\nu \right)\propto S\left(q,\;\nu \right)/n_{B}\left(\nu \right)$, where $n_{B}\left(\nu \right)$ is the Bose factor and $S\left(q,\nu \right)$ is the dynamical structural factor, furnishing the distribution of dynamical modes over frequency *ν* at a given wave vector (*q*). Relaxation processes on different time scales appear as distinct peaks in $\chi^{\prime \prime }\left(q,\;\nu \right)$ with associated relaxation times $\tau =1/\left(2\pi \nu_{\mathrm{peak}}\right),$^20,44^ where $\nu_{\mathrm{peak}}$ is the peak frequency and τ is roughly the time required for the water molecules to diffuse a distance of ∼*2π*/*q*. $\chi^{\prime \prime }\left(q,\;\nu \right)$ measured at different wave vectors for the different samples presented in Figs. 2(a)–2(f). As can be seen, for each sample, the peak in $\chi^{\prime \prime }\left(q,\;\nu \right)$ shifts to higher frequency with increasing *q*, as it takes less time for water molecules to diffuse a shorter distance (2π/*q*). The MD-derived neutron spectra of bulk water and hydration water in the protein powders are also presented in the figures, overlaid onto the experimental data. A quantitative agreement is observed, validating the water dynamics obtained via MD.

**FIG. 3.**
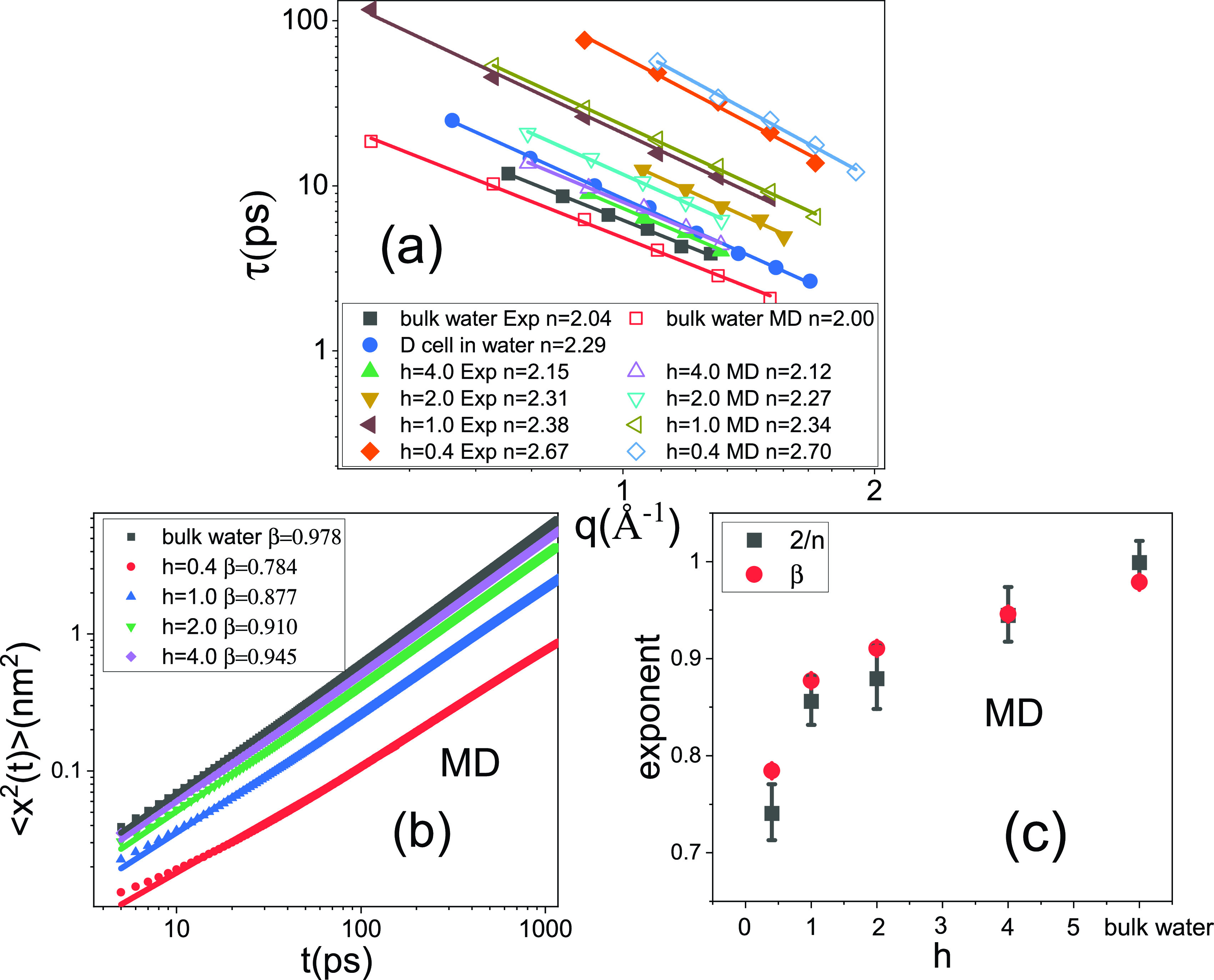
(a) *q* dependence of the characteristic relaxation time τ of the hydration water at different hydration levels (*h* = 4.0, *h* = 2.0, *h* = 1.0, and *h* = 0.4), bulk water and water in *E. coli*. The values of τ are derived by fitting the Cole–Cole distribution function to the experimental and MD-derived $\chi^{\prime \prime }\left(q,\upsilon \right)$ spectra. Solid lines represent the power law fits, i.e., *τ* ∼ *q^−n^*, and the values of *n* are shown in the figure. (b) Mean-squared atomic displacement $\left\langle x^{2}(t)\right\rangle$ derived from the MD simulation. The lines represent power law fits in the time window from 5 ps to 1 ns. (c) The sub-diffusive exponents *β*(red dots) obtained by direct power-law fits of the MD-derived $\left\langle x^{2}(t)\right\rangle$ from Fig. 3(b), the corresponding MD values of *n* are taken from Fig. 3(a), and the error bars of *β* are negligible as compared to the size of the symbols. Thus, Fig. 3(c) reveals the connection of *β* =2/*n*.

For each spectra measured at a given *q*, the characteristic relaxation time, τ, can be obtained by fitting the Cole–Cole distribution function to the neutron spectra:^36,45^

$$\chi^{\prime \prime }\left(q,\nu \right)=\chi_{0}\frac{{\left(2\pi \nu \tau \right)}^{\left(1-\alpha \right)}\text{cos}(\frac{\pi \alpha }{2})}{1+2{\left(2\pi \nu \tau \right)}^{\left(1-\alpha \right)}\text{sin}\left(\frac{\pi \alpha }{2}\right)+{\left(2\pi \nu \tau \right)}^{2(1-\alpha )}},$$ (1)

where τ, α, and $\chi_{0}$ are fitting parameters. An example is given for the *E. coli* cells in Fig. 2(f). The resulting values of τ are presented in Fig. 3(a). As can be seen, at any given *q*, τ of water increases by about an order of magnitude in going from *h* = 4.0 to *h* = 0.4, i.e., water is slowed down by a factor of 10. More importantly, *τ* follows a power-law dependence on *q*, $\tau ∼q^{-n}$, with *n* decreasing from 2.7 to 2.1 accordingly [Fig. 3(a)]. As τ furnishes the time required for the water molecules to diffuse a distance ∼2π/*q*, the mean-squared atomic displacement should scale with time as, ⟨x^2^(t)⟩ ∼ (1/*q*)^2^ ∼ *t*^2/n^. Therefore, the diffusion power law *β* in $\langle x^{2}\left(t\right)\rangle ∼t^{\beta }$ can be estimated as 2/*n*. This scheme of estimation of *β* is quantitatively validated by the MD simulations in Fig. 3(c), where *β* is obtained by fitting the time dependence of the MD-derived $\left\langle x^{2}\left(t\right)\right\rangle$ in the window from 5 ps to 1 ns [see Fig. 3(b)] at a given hydration level, and *n* is derived from the power-law fits to τ as a function of *q*, where τ is acquired by fitting Eq. (1) to the MD-derived neutron spectra [Fig. 3(a)].

Moreover, for all biological samples, the values of *n* exceed 2, and they decrease when increasing hydration [Fig. 3(a)]. Hence, one can conclude that water in all biological samples conduct sub-diffusive motions, which gradually diminishes when increasing the water content.

**FIG. 4.**
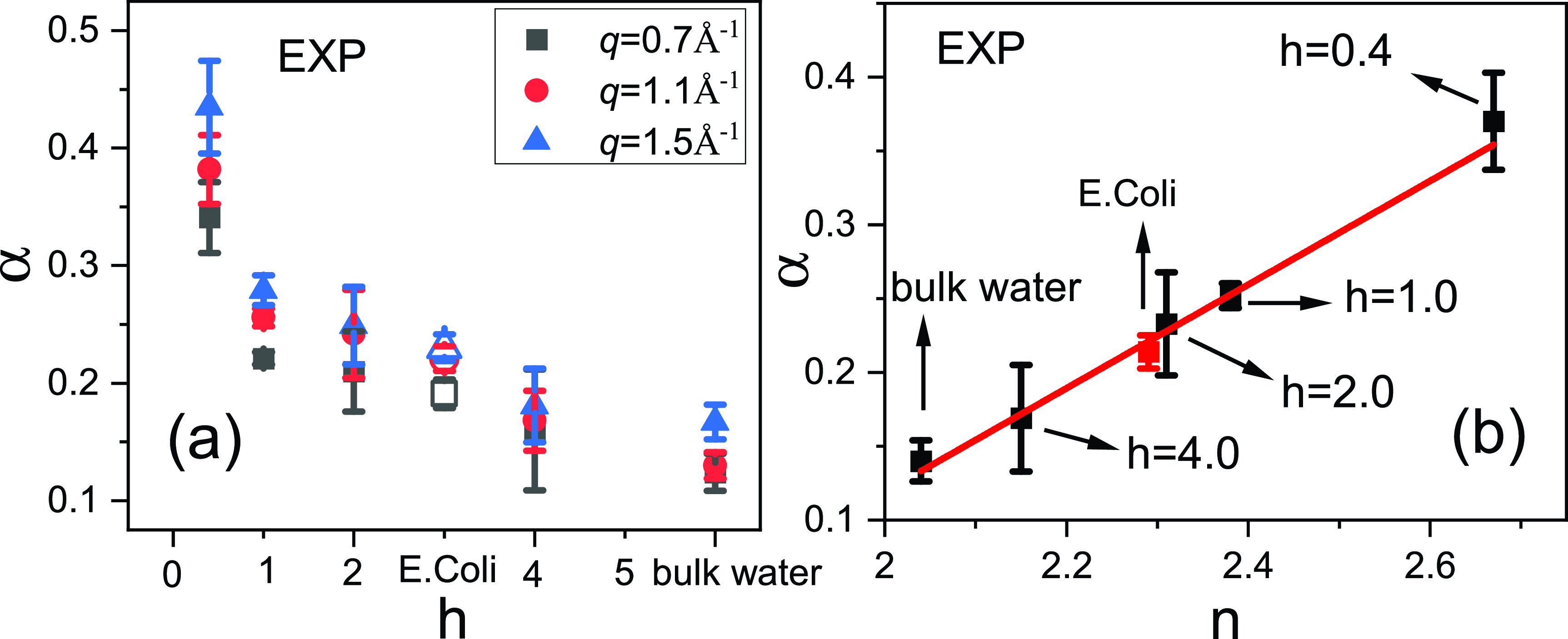
(a) The values of *α* derived from the fitting of the experimental neutron spectra for different systems at three different values of *q*, the empty symbols represent the *α* values in *E. coli* cells. (b) The relationship between the experimental *α* (averaged over the *q* from 0.7 Å^−1^ to 1.5 Å^−1^ for different samples) and *n*, where the red point corresponds to water in *E. coli* cells and the red line is a guide for the linear relation between the two parameters.

Another parameter derived from the fitting is the stretching parameter, α, which connects to the distribution of the mobility, where a larger value of *α* corresponds to a more heterogeneous distribution.^36,46^ As seen in Fig. 4(a), despite slight variations with *q*, *α* increases with decreasing water content, indicating that as the system is less hydrated, the distribution of water mobility becomes broader. Furthermore, plotting *α* averaged over *q* (between 0.7 Å^−1^ to 1.5 Å^−1^) vs *n* for different samples, as shown in Fig. 4(b), reveals a positively correlated linear relation between these two parameters. This linear correlation indicates that water molecules with a broader distribution of mobility will exhibit a more sub-diffusive character. In other words, by measuring the sub-diffusive behavior, one can quantitatively characterize the underlying dynamical heterogeneity. This is consistent with our previous work,^20^ which describes the sub-diffusion of water in hydrated protein powders as a simple continuous time random walk model (CTRW). In this model, particles are assumed to conduct continuous jumps, and the residence time between adjacent jumps obeys a broad power-law distribution, which determines the sub-diffusive exponent of the particles.

Moreover, when carefully comparing the fitting results of the living cell with those of hydration water in protein samples [see Fig. 4(b) and Table I], one can see that τ of the water in the living cells closely approximates that of protein-surface water at the hydration level of *h* = 4.0. However, the values of *n* and α of the living cells are closer to those of the sample with *h* = 2.0. This comparison reveals that, although the mobility of water in the living cell is closer to that of the hydration water at *h* = 4.0 (as they have the same amount of water content), the dynamical heterogeneity in the living cell is much greater and approximates that of a protein sample with much lower water content. A living cell is composed of all different biomolecules (protein, DNA, RNA, and lipid membranes), and thus provides a more complex environment for water.

**TABLE I.** The comparison of τ(q), *n*, and α in protein samples at *h* = 2.0 and *h* = 4.0 and in *E. coli* cells derived from neutron experimental spectra. The values of *α* are averaged over the *q* from 0.7 Å^−1^ to 1.5 Å^−1^ and the values of *n* are obtained from Fig. 3(a) by power law fits.

	τ (*q* = 0.9 Å^−1^) (ps)	τ (*q* = 1.1 Å^−1^) (ps)	τ (*q* = 1.3 Å^−1^) (ps)	*n*	α
*h* = 2.0	16.693	12.465	7.485	2.31	0.233
*h* = 4.0	8.953	6.344	4.017	2.15	0.169
*E. coli*	9.999	7.370	3.987	2.29	0.214

## IV. INSIGHTS FROM SIMULATIONS

**FIG. 5.**
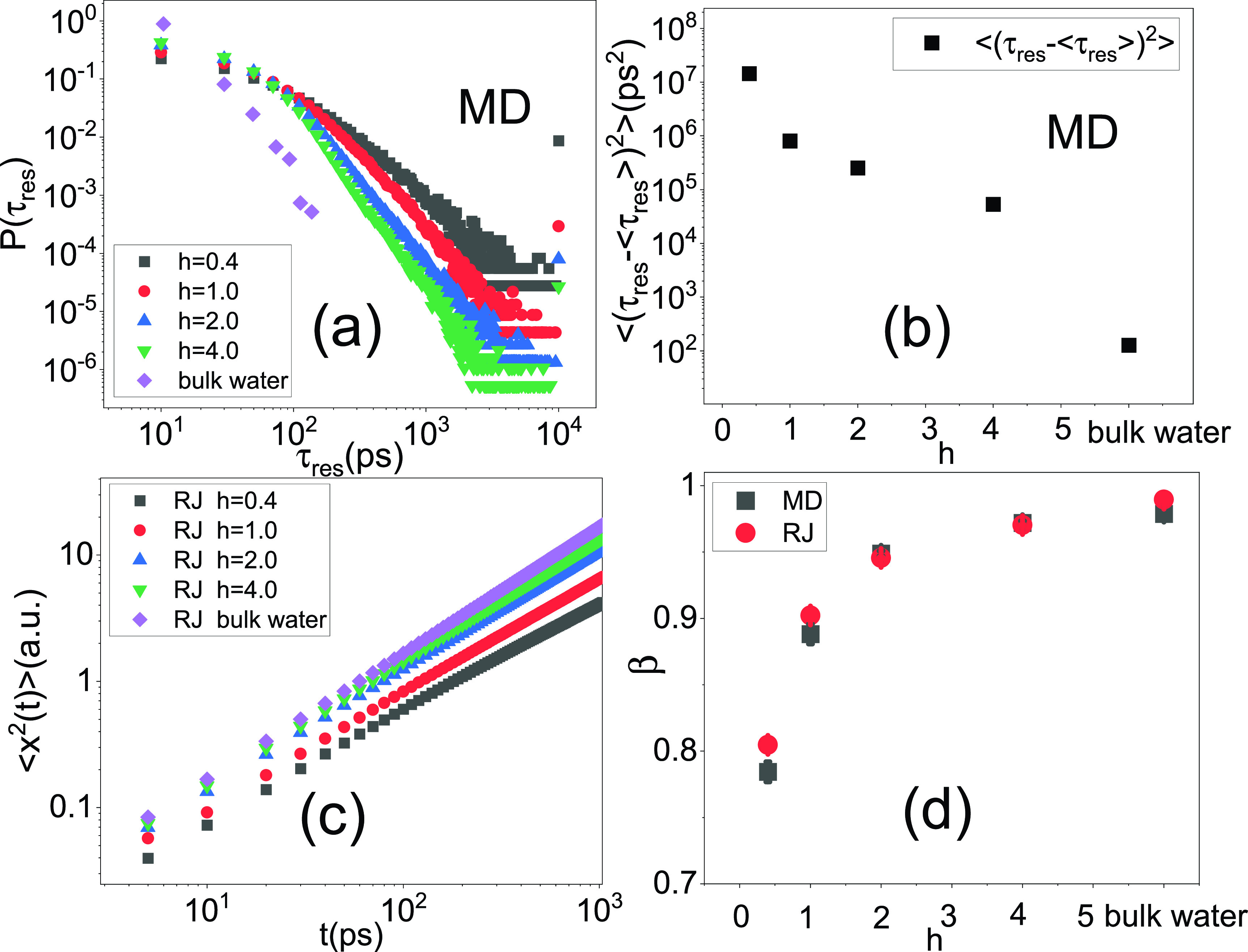
(a) The MD-derived distribution of residence time, $P(\tau_{\mathrm{res}})$, for different systems. (b) The secondary moments of $\tau_{\mathrm{res}}$, defined as the squared-deviation of the residence time from its mean, i.e., ⟨($\tau_{\mathrm{res}}$ −⟨$\tau_{\mathrm{res}}$⟩)^2^⟩. (c) $\left\langle x^{2}(t)\right\rangle$ derived from the coarse-grained random jump model. (d) The sub-diffusive exponent *β* obtained by power law fits to $\left\langle x^{2}(t)\right\rangle$ derived from MD [Fig. 3(b)] and from the coarse-grained RJ model [Fig. 5(c)] in the same time window (5 ps to 1 ns).

As described by the CTRW model, the distribution of microscopic mobility of water molecules should directly determine the sub-diffusive power law, *β*, such that a broader distribution will lead to more prominent sub-diffusion.^20^ To examine this toy model, one needs to define the microscopic mobility of individual water molecules. We tracked the molecular dynamics simulation trajectory of each water molecule and defined microscopic mobility as the residence time required for the water molecule to move a distance of 3.5 Å^33,36^ (see the details in the supplementary material), noted as $\tau_{\mathrm{res}}$. Such a distance is chosen since it corresponds to the first minimum in the radial distribution function between oxygen atoms in bulk and hydration water, representing the thickness of water's first coordination shell.^33^ The resulting distribution of $\tau_{\mathrm{res}}$, $P(\tau_{\mathrm{res}})$, for hydration water at different hydration levels, *h*, and for bulk water is presented in Fig. 5(a). Indeed, $P(\tau_{\mathrm{res}})$ increasingly broadens upon reducing *h*, in agreement with the more prominent sub-diffusive motions found by experiment and simulation [see Figs. 3(a) and 3(c)]. Furthermore, it qualitatively confirms the prediction of the CTRW model. A more quantitative characterization of the width of the distribution of $\tau_{\mathrm{res}}$ is presented in Fig. 5(b), which displays the second moment of $P(\tau_{\mathrm{res}})$, i.e., the mean-squared deviation of the residence time from its mean, ⟨($\tau_{\mathrm{res}}$ − ⟨$\tau_{\mathrm{res}}$⟩)^2^⟩. As can be seen, ⟨($\tau_{\mathrm{res}}$ −⟨$\tau_{\mathrm{res}}$⟩)^2^⟩ increases by orders of magnitude when decreasing hydration levels from *h* = 4.0 to 0.4.

To quantitatively examine the CTRW model, we performed a coarse-grained numerical simulation of random jumps, referred to as RJ. Briefly, a particle is assumed to perform a one-dimensional random jump, where each jump takes a constant distance (*l_0_*) with equal probability to randomly go forwards or backwards, while the residence time between two adjacent sites is randomly taken from $P(\tau_{\mathrm{res}})$ in Fig. 5(a). The values of ${\langle x}^{2}\left(t\right)\rangle$ obtained from this RJ simulation are presented in Fig. 5(c) and the corresponding values of the sub-diffusive exponent *β* are shown in Fig. 5(d). As can be seen in Fig. 5(d), the values of *β* derived from the RJ modeling are in quantitative agreement with those obtained from the all-atom MD simulations, demonstrating that it is the distribution of the microscopic mobility that causes the sub-diffusive motions of interfacial water in all protein samples at varying hydrations from *h* = 0.4 to 4.0.

**FIG. 6.**
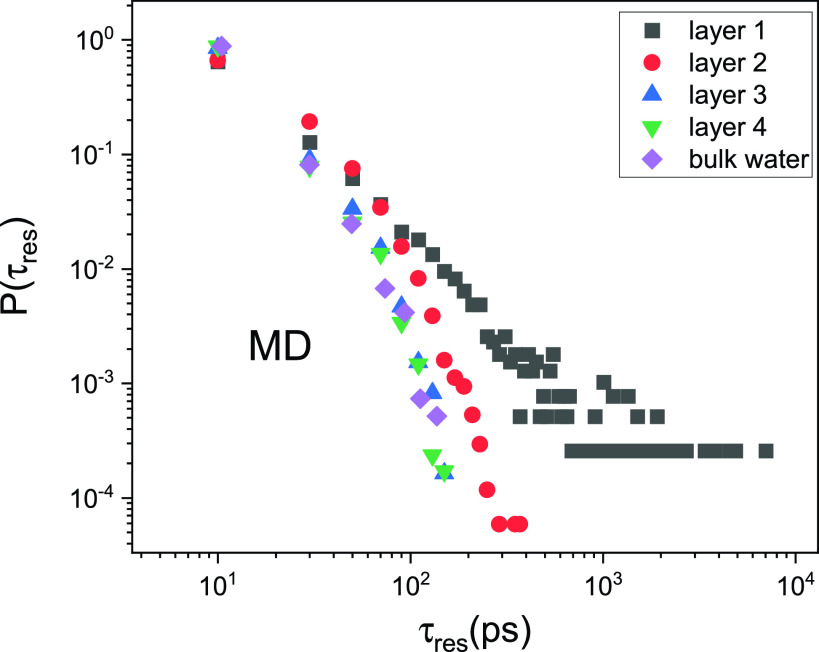
The distribution of residence times, $P\left(\tau_{\mathrm{res}}\right)$, at each hydration layer in the sample at *h* = 4.0 and compared to that in the pristine bulk water.

The broad distribution of residence times likely results from the complex structural and chemical features of the protein surface. However, of interest is also how the heterogeneity of *τ*_res_ varies layer-by-layer on the protein surface. Here, taking *h* = 4.0 as an example, we analyzed *P*(*τ*_res_) of water at each specific hydration layer, whose thickness is 3.5 Å, and the results are presented in Fig. 6 (detailed procedure to obtain *τ*_res_ at each hydration layer can be found in the supplementary material). As can be seen, the mobility of the first layer of water exhibits the most heterogeneity, and this effect extends to the second hydration layer, whereas the mobility of water beyond the second layer is approximately the same as in bulk water. Hence, bulk-like characteristics start to be observed on the protein surface from the third layer, which is surprisingly consistent with similar observations on the behavior of hydration water at the surface of graphene oxide,^47^ indicating this might be a general phenomenon for various interfaces. Furthermore, as the first two layers of water account for ∼1.2 g water/gram protein, one can estimate that 40% water at *h* = 2.0 is bulk-like water, and this number is 70% for *h* = 4.0. As the dynamical behavior of water in *E. coli* cells falls in between that of *h* = 2.0 and 4.0 (see Table I), one can deduce that between 40% and 70% water in the cell is bulk-like water. This is slightly lower than the values (∼85%) estimated by fitting the corresponding neutron spectra by a sum of multiple Lorentzian functions with each representing one type of dynamical components, e.g., translational or rotational motions of bulk or hydration water.^26^ The difference might result from the fact that the Lorentzian functions are too simple a model to capture the complexity and heterogeneity of the dynamics in hydration water involved. Moreover, as revealed by Ref. 32, which combines time-resolved infrared and dielectric-relaxation spectroscopies, only ∼12% of water is slowed down in *E. coli*, much less than the values estimated here. The two techniques used in Ref. 32 mostly measure the orientational motion of water, while the neutron is highly sensitive to translation. As compared to the orientational motion, the water translation is altered much more in terms of the retardation factor and the spatial extent by the presence of the biomolecules.^38^ As a result, a much greater amount of perturbed water might be observed when measured using neutron as compared to that probed by the time-resolved infrared and dielectric-relaxation spectroscopies. It would be very interesting to combine neutron scattering with the time-resolved vibrational spectroscopy to examine how different dynamical modes of the biological water vary among different cells.

## V. CONCLUSION

Neutron scattering, in particular, the technique of quasi-elastic neutron spectroscopy, was successfully employed here to characterize the nature of the translational dynamics of water on hydrated perdeuterated Cytochrome P450 protein at different hydration levels ranging from hydrated powder to a concentrated solution, and compare it with that in a living cell system, perdeuterated *E. coli* cells. In all samples, water exhibits sub-diffusive motions, however it becomes less prominent with increasing water content. Complementary simulations reveal that the sub-diffusive character arises from the distribution of microscopic mobility of water molecules, and that a broader distribution of mobility leads to more prominent sub-diffusion. Moreover, the MD simulations revealed a different level of heterogeneity in different water hydration layers, with bulk-like behavior observed beyond the second layer. Finally, a comparison of the results for water in the *E. coli* cells with that in the concentrated protein solution of similar water content revealed that, although the timescale of water dynamics in both systems is very similar, the heterogeneity of the water motions in the cell is much greater.

## SUPPLEMENTARY MATERIAL

See the supplementary material for details of sample preparation of deuterated *E. coli*, deuterated protein synthesis, neutron scattering experiment, MD simulations, and the calculation method.

## AUTHORS' CONTRIBUTIONS

The manuscript was written through contributions of all authors.

## Data Availability

The data that support the finds of this study are available from the corresponding author upon reasonable request.
